# Twitter-Derived Social Neighborhood Characteristics and Individual-Level Cardiometabolic Outcomes: Cross-Sectional Study in a Nationally Representative Sample

**DOI:** 10.2196/17969

**Published:** 2020-08-18

**Authors:** Dina Huang, Yuru Huang, Sahil Khanna, Pallavi Dwivedi, Natalie Slopen, Kerry M Green, Xin He, Robin Puett, Quynh Nguyen

**Affiliations:** 1 Department of Epidemiology and Biostatistics University of Maryland School of Public Health College Park, MD United States; 2 A. James Clark School of Engineering University of Maryland College Park, MD United States; 3 Department of Behavioral and Community Health University of Maryland School of Public Health College Park, MD United States; 4 Maryland Institute for Applied Environmental Health University of Maryland School of Public Health College Park, MD United States

**Keywords:** neighborhood study, cardiometabolic outcomes, Twitter

## Abstract

**Background:**

Social media platforms such as Twitter can serve as a potential data source for public health research to characterize the social neighborhood environment. Few studies have linked Twitter-derived characteristics to individual-level health outcomes.

**Objective:**

This study aims to assess the association between Twitter-derived social neighborhood characteristics, including happiness, food, and physical activity mentions, with individual cardiometabolic outcomes using a nationally representative sample.

**Methods:**

We collected a random 1% of the geotagged tweets from April 2015 to March 2016 using Twitter’s Streaming Application Interface (API). Twitter-derived zip code characteristics on happiness, food, and physical activity were merged to individual outcomes from restricted-use National Health and Nutrition Examination Survey (NHANES) with residential zip codes. Separate regression analyses were performed for each of the neighborhood characteristics using NHANES 2011-2016 and 2007-2016.

**Results:**

Individuals living in the zip codes with the two highest tertiles of happy tweets reported BMI of 0.65 (95% CI –1.10 to –0.20) and 0.85 kg/m^2^ (95% CI –1.48 to –0.21) lower than those living in zip codes with the lowest frequency of happy tweets. Happy tweets were also associated with a 6%-8% lower prevalence of hypertension. A higher prevalence of healthy food tweets was linked with an 11% (95% CI 2% to 21%) lower prevalence of obesity. Those living in areas with the highest and medium tertiles of physical activity tweets were associated with a lower prevalence of hypertension by 10% (95% CI 4% to 15%) and 8% (95% CI 2% to 14%), respectively.

**Conclusions:**

Twitter-derived social neighborhood characteristics were associated with individual-level obesity and hypertension in a nationally representative sample of US adults. Twitter data could be used for capturing neighborhood sociocultural influences on chronic conditions and may be used as a platform for chronic outcomes prevention.

## Introduction

The neighborhood environment has been recognized as an important determinant of health. Previous studies have identified associations between neighborhood characteristics and health behaviors [1,2], chronic conditions [3,4], and mental health outcomes [5,6]. Access to healthy food, proximity to parks, recreational facilities, and neighborhood walkability are protective factors of obesity, diabetes, and hypertension [7-10]. Conversely, neighborhood disadvantage and neighborhood-level stressors are associated with higher prevalence of obesity and hypertension [3,11-13].

In addition to the physical environment, social contextual factors are also associated with a variety of health outcomes. The Roseto Effect describes the phenomenon in which members of a close-knit community experience a lower heart rate than members of a neighborhood community and is an example of the potential influence of the social environment [14]. Research has shown that greater community happiness is associated with decreased prevalence of obesity, hypertension, and suicide, as well as increased life expectancy [15-17]. Greater social cohesion has been linked with lower hypertension [7,11], and social capital has been linked with lower prevalence of obesity, hypertension, and mental health conditions [18,19]. Social media, such as Twitter, can serve as a data source to characterize the social neighborhood environment. Previous studies using Twitter data have validated the approach for assessing dietary choices, measuring happiness, and examining community levels of physical activity [20-22]. Unlike traditional indicators of neighborhood environment, Twitter indicators reflect an individual’s perception and attitude towards the neighborhood environment, as well as an individual’s use of neighborhood resources [23]. Traditional neighborhood studies mainly rely on time-consuming neighborhood data collection within limited geographical areas. In comparison, Twitter-derived indicators as proxy measures for neighborhood factors provide low-cost opportunities to conduct neighborhood studies at the national level and to study the association between geographic factors and health outcomes. We hypothesize that neighborhood-level factors, as estimated by aggregating information from tweets, influence individual-level health.

### Underlying Mechanism

According to social learning theory, learning occurs in a social context [24]. Social context influences individual health behaviors through reciprocal interactions between people, as well as between people and the environment, through observational learning of modeled behaviors, self-initiated reinforcement, or external positive or negative reinforcement, and socially communicated expectations of particular health behaviors [24]. For instance, communities that tweet more about physical activity may culturally support such activity more than other communities, thus reinforcing the decision of a given resident to engage in similar activity. Communities might also differ in the foods they prefer; therefore, utilizing Twitter data, we can estimate food preferences and norms and determine whether these relate to health outcomes on an individual level.

### Study Aim and Hypothesis

In this study, we utilized Twitter-derived characteristics, including happiness, food, and physical activity, as social neighborhood predictors. We assessed the associations between the Twitter-derived characteristics and cardiometabolic outcomes, including obesity, diabetes, and hypertension, using a nationally representative sample from the National Health and Nutrition Examination Survey (NHANES). We hypothesized that people living in zip codes with high levels of Twitter-derived neighborhood happiness, healthy diet, and physical activity have lower mean BMI and lower prevalence of obesity, diabetes, and hypertension, respectively.

## Methods

### Study Population and Outcomes

Individual-level health data were obtained from the NHANES 2007-2008, 2009-2010, 2011-2012, 2013-2014, and 2015-2016. We received approval to access the restricted, geocoded data from the National Center for Health Statistics (NCHS) Restricted Data Center (RDC). NHANES data and Twitter-derived predictors were merged via zip code linkages by an NCHS analyst. Zip codes were masked after data linkage. All statistical analyses were performed at the Maryland Federal Statistical Research Data Center, and all output was reviewed by an RDC staff member to avoid information disclosure. We followed the RDC confidentiality and disclosure review policies to protect the confidentiality of the NCHS study participants.

NHANES data consist of interview data (demographic, socioeconomic, and health-related questions) and examination data (physiological checks as well as laboratory tests). Data collection for NHANES was approved by the NCHS Research Ethics Review Board (ERB). Analysis of deidentified data from the survey is exempt from the federal regulations for the protection of human research participants. Analysis of restricted data through the NCHS RDC is also approved by the NCHS ERB. The study was approved by the University of Maryland Institutional Review Board (IRB).

We examined the following cardiometabolic outcomes: BMI, obesity, diabetes, and hypertension. NHANES measured weight and height data were used to calculate BMI. Obesity was defined as BMI≥30 kg/m^2^. BMI and obesity are interdependent outcomes. Given BMI is a continuous variable, it provides more statistical power to detect differences, enabling readers to assess how much Twitter-derived variables might shift the distribution of BMI values. However, obesity is a clinically important health outcome. We present analyses with both to provide a more comprehensive examination of associations between Twitter-derived community variables and health outcomes. Hypertension was defined as having elevated blood pressure or self-report of taking medications for hypertension. A mean systolic blood pressure >130 mm Hg or mean diastolic blood pressure >80 mm Hg was defined as elevated blood pressure [25]. Diabetes was defined as having a glycohemoglobin (%) value ≥6.5% or self-reported diagnoses of diabetes [26].

We included both individual-level and zip code-level covariates to account for confounding. Individual-level covariates from NHANES included age, sex, race/ethnicity, and annual household income. Zip code level characteristics included the following: percent of non-Hispanic white, median household income, population density, and median age obtained from the 2011-2015 American Community Survey 5-year estimates [27]. To avoid disclosure of zip codes, we replaced continuous percent non-Hispanic white, median age, and population density with the corresponding median value in each 20-quantile group. We replaced continuous median household income with the corresponding log-transformed median value for each 20-quantile group as requested by the RDC.

### Twitter-Derived Social Neighborhood Characteristics

A random 1% of the geotagged tweets that are publicly available were collected through Twitter’s streaming application programming interface (API) from February 2015 to March 2016. Geotagged tweets have the latitude and longitude coordinates of the location from which they were sent. We collected 79,848,992 geotagged tweets across the contiguous United States (including Washington, DC) and identified 603,363 unique Twitter users. Duplicated tweets and tweets identified as job postings through hashtags were removed. Each tweet was linked to the corresponding zip code through spatial join using Python (Python Software Foundation) [28].

**Figure 1:**
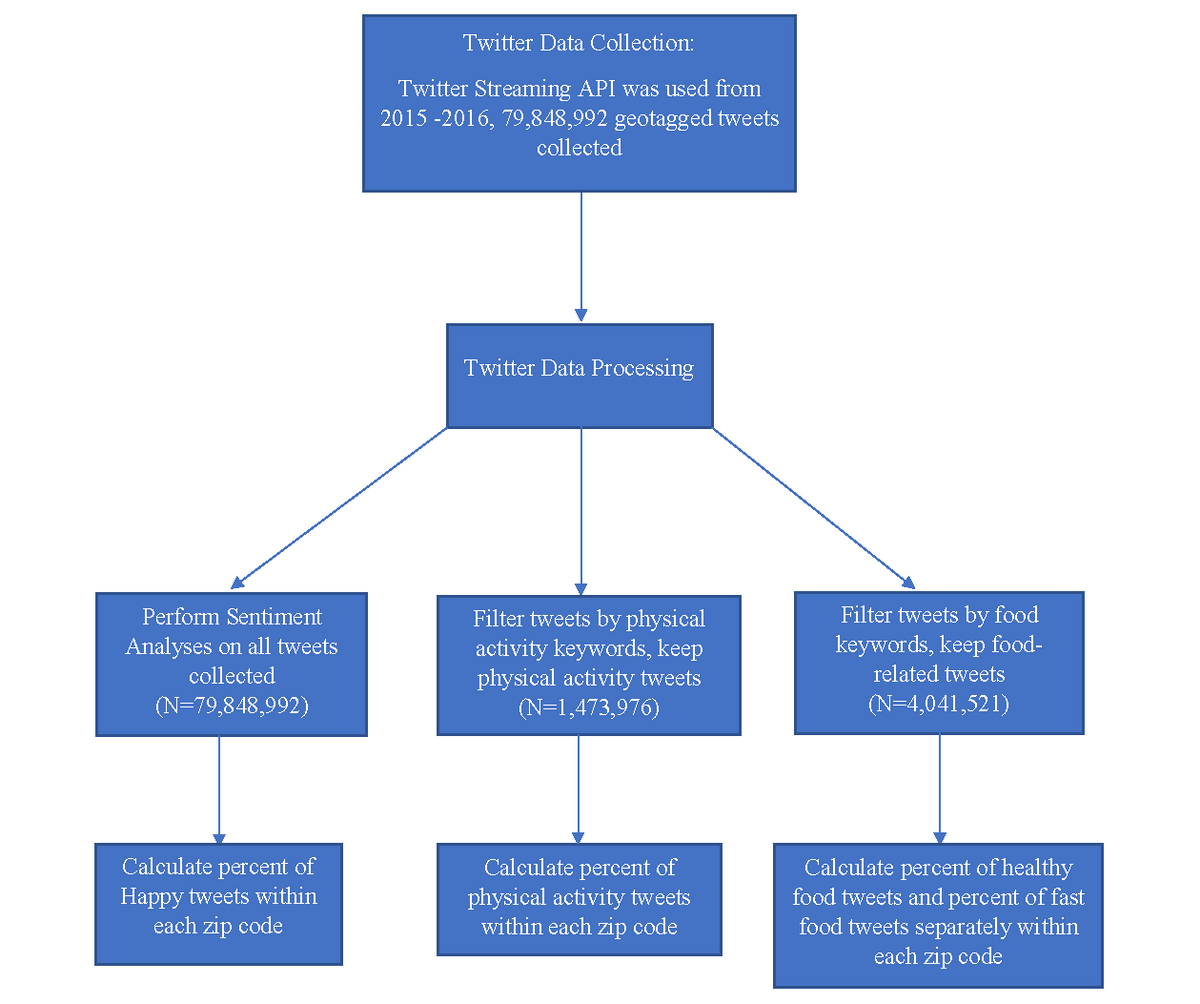
Twitter data collection and construction of Twitter characteristics.

Detailed information on the construction and validation of Twitter characteristics can be found in a previously published article [20]. Figure 1 is a flowchart of Twitter data collection and the construction of Twitter characteristics. Here, we briefly summarized the process to construct Twitter characteristics. We implemented sentiment analysis with the Machine Learning for Language Toolkit (MALLET) [29] to compute the happiness score (range from 0 to 1) for each tweet. We included diverse sources of training data such as Sentiment140 [30], Sanders Analytics [31], and Kaggle [32]. A binary variable of happiness was created for each tweet based on the MALLET score, where a rating >0.8 was defined as happy. The cut-off point of 0.8 reached the highest accuracy of classifying happy tweets and maintained the same prevalence of happy tweets identified in the human-labeled dataset [20]. After identifying each tweet as “happy” versus “not happy,” we calculated the percent of happy tweets within each zip code [33].

For food analysis, we created a list of over 1430 popular food words from the US Department of Agriculture’s National Nutrient Database [34]. Fruits, vegetables, nuts, and lean proteins were labeled as healthy food (n=340), and fast food labels were also used (n=154). We identified 4,041,521 food-related tweets and filtered each tweet by items on the food list to categorize them as mentions of healthy or fast food. The percentages of healthy and fast food tweets were calculated at the zip code level.

Similar to our food analysis, we created a list of physical activities using the published list of physical activity terms collected from physical activity questionnaires, a compendium of physical activities, and popular fitness programs [35-37]. A total of 376 items were gathered and included activities from gym exercise, sports, recreational activities, and household chores. Expressions such as “running late” and “walk away” were excluded. To avoid including tweets about passively watching sports, we excluded tweets with the verbs “watch/watching/watches/watched” and “attend/attends/attending/attending” and only included team sports tweets when there was the word “play/plays/playing/played.” We collected 1,473,976 geotagged tweets associated with physical activity. The percentage of physical activity tweets was aggregated at the zip code level.

To comply with RDC confidentiality and disclosure review policies, we were unable to use continuous percent Twitter characteristics at the zip code level, since the data may serve as geo-identifiers. We categorized Twitter characteristics at the zip code level into tertiles (high, medium, low) as predictors.

### Statistical Analysis

We implemented separate regression analyses for each of the outcomes. Linear regression was used for continuous outcomes such as BMI; Poisson regression was used for binary outcomes including obesity, diabetes, and hypertension, to estimate prevalence ratios [38]. All models controlled for individual-level demographics and zip code-level characteristics. We analyzed health outcomes for the NHANES 2011-2016 subcohort, which is closer in time to the Twitter data (2015-2016). As a supplement, we analyzed NHANES data from the most recent five survey cycles from 2007 to 2016 (described below as the “full cohort”) to obtain a sample with a higher diversity of zip codes (2116 zip codes in the full cohort and 1384 zip codes for the subcohort). A 10-year Mobile Examination Center (MEC) weight was used for NHANES data from 2007 to 2016, and a 6-year MEC weight was used for NHANES data from 2011 to 2016 [39,40]. Analyses were performed in Stata MP15 (StataCorp LP).

## Results

### Descriptive Statistics

Descriptive statistics are shown in Tables 1 and 2. The zip code–level Twitter characteristics were calculated for all zip codes in the United States. Of these, 19% were happy (n=29,606), 2.2% mentioned physical activity, and 5.0% mentioned food. There were fewer tweets about healthy foods (1.0%) and fast food (0.3%). Examples of each Twitter-derived characteristic are listed in Table 3.

For the full cohort, the mean age was 47 years, and 15,040 of 29,201 participants (51.9%) were female. Reported participant race and ethnicity included 12,113 (66.6%) non-Hispanic white, 7627 (14%) Hispanic, 6179 (11%) non-Hispanic black, and 3282 (8%) identified as other races. The mean BMI was 29 kg/m^2^, and the prevalence of obesity was 36.5% (10,478 participants). The mean glycohemoglobin level was 5.6%, and the prevalence of diabetes was 12.1% (n=4603). Hypertension was reported in 14,336 participants (48.1%). Individual demographic characteristics in the subcohort were similar to those in the full cohort.

**Table 1:** Descriptive characteristics for Twitter social neighborhood characteristics.

Zip code level Twitter characteristics	Number of zip codes with Twitter characteristics	Mean percentage (SE)
Happy tweets	29,606	19.0 (0.06)
Tweets about physical activity	29,604	2.2 (0.02)
Tweets about food	24,177	5.0 (0.03)
Tweets about healthy food	24,173	1.0 (0.02)
Tweets about fast food	24,174	0.3 (0.01)

**Table 2:** Descriptive characteristics for individual characteristics from the National Health and Nutrition Examination Survey.

Individual-level characteristics		NHANES^a^ 2007-2016^b^		NHANES 2011-2016^c^	
		Total participants, n	Mean, % (SE)	Total participants, n	Mean, % (SE)
Age (years), mean (SE)		29,201	47.3 (0.25)	17,048	47.6 (0.36)
Female, % (SE)		15,040	51.9 (0.28)	8803	52.0 (0.40)
Married, % (SE)		14,836	55.0 (0.72)	8534	54.2 (1.02)
**Race/Ethnicity, % (SE)**					
	Black, non-Hispanic, % (SE)	6179	11.4 (0.82)	3830	11.4 (1.17)
	White, non-Hispanic, % (SE)	12,113	66.6 (1.62)	6376	65.4 (2.13)
	Hispanic, % (SE)	7627	14.3 (1.11)	4156	14.8 (1.45)
**Education, % (SE)**					
	Less than high school	7579	17.2 (0.70)	3942	15.5 (0.97)
	High school	6596	22.1 (0.55)	3708	20.9 (0.71)
	Some college	8366	31.4 (0.52)	5119	32.5 (0.76)
	College or greater	6621	29.3 (1.02)	4262	31.2 (1.45)
**Total annual household income (US$), % (SE)**					
	<20,000	6247	15.0 (0.61)	3593	14.9 (0.85)
	20,000-55,000	11,518	37.1 (0.67)	6453	36.1 (0.97)
	55,000-75,000	2965	12.6 (0.44)	1709	12.3 (0.59)
	75,000-100,000	2503	11.4 (0.38)	1437	10.8 (0.41)
	≥100,000	4399	23.9 (1.09)	2861	25.9 (1.61)
BMI (kg/m^2^), mean (SE)		28,818	28.9 (0.09)	16,830	29.1 (0.12)
Obesity, % (SE)		10,478	36.5 (0.54)	6144	37.6 (0.76)
Hemoglobin A_1c_, % (SE)		27,775	5.6 (0.01)	16,280	5.6 (0.01)
Diabetes prevalence, % (SE)		4603	12.1 (0.32)	2741	12.6 (0.42)
Hypertension, % (SE)		14,336	48.1 (0.59)	8411	48.8 (0.77)

^a^NHANES: National Health and Nutrition Examination Survey.

^b^Descriptive statistics were weighted using the Mobile Examination Center 10-year weight.

^c^Descriptive statistics were weighted using Mobile Examination Center 6-year weight.

**Table 3:** Examples of each Twitter characteristic^a^.

Example number	Happy tweets	Fast food tweets	Healthy food tweets	Physical activity
Example 1	“I am so blessed that my family is healthy – it is all it matters!”	“I just left pizzahut with my mother!”	“collard greens are so delicious”	“gotta get up and workout in a couple hours hopefully I can get up 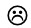 ”
Example 2	“Me & my bestie celebrating her bachelorette trip. We are having a blast!”	“The perfect afternoon work spot @starbucks”	“Today woke up at 8 am to eat a kale salad”	“I just finished running 6.02 miles in 50m:44s”
Example 3	“Wednesday night with the best people!”	“Taco Bell run”	“I cooked for lunch today! Brown rice with roast beef, broccoli, and green beans – yummm!”	“A fun seven-mile hike at Shenandoah”
Example 4	“Brunch after the hike!!!#foodporn”	“Chipotle line mad long but I am not leaving!”	“Turkey, broccoli, spinach, and tomatoes! This is breakfast yay”	“hiked to the summit of a mountain today!”

^a^Example tweets were slightly modified to mask the original tweets. Specific time, location, and names were changed to avoid identity disclosure.

### Regression Results

Zip code-level happiness was associated with lower mean BMI, as well as a lower prevalence of hypertension (Table 4). Comparing individuals living in the medium (second tertile) and the highest (third tertile) to the lowest level (first tertile) of happy tweets, mean BMI decreased by 0.65 kg/m^2^ (95% CI –1.10 to –0.20) and 0.85 kg/m^2^ (95% CI –1.48 to –0.21), respectively. The prevalence of hypertension was lower by 8% (prevalence ratio [PR] 0.92; 95% CI 0.86 to 0.98) and 6% (PR 0.94; 95% CI 0.88 to 1.00) in the medium and highest tertiles versus the lowest tertile (Table 4). Associations between happy tweets and obesity and diabetes bordered on statistical significance in the subcohort analyses, but were statistically significant in the full cohort analyses.

High levels of Twitter-derived physical activity were associated with a lower prevalence of hypertension. In a comparison of individuals living in zip codes in the medium and highest levels of physical activity tweets to those with the lowest level, hypertension decreased by 8% (PR 0.92, 95% CI 0.87 to 0.98) and 10% (PR 0.90, 95% CI 0.85 to 0.96), respectively. Physical activity tweets were not associated with BMI, obesity, and diabetes.

Healthy food tweets were linked to BMI, obesity, and hypertension. Individuals living in zip codes with medium and high levels of healthy food tweets had mean BMI values that were 0.73 kg/m^2^ lower (95% CI –1.39 to –0.07) and 1.02 kg/m^2^ lower (95% CI –1.39 to –0.07). The prevalence of obesity was 5% (PR 0.95, 95% CI 0.86 to 1.04) and 11% lower (PR 0.88, 95% CI 0.79 to 0.98) and the prevalence of hypertension was 6% (PR 0.94, 95% CI 0.88 to 1.00) and 1% (PR 0.99, 95% CI 0.91 to 1.06) lower. Fast food tweets were not associated with BMI, obesity, hypertension, and diabetes. Table 4 shows the number of study participants with given characteristics.

In supplemental analyses using NHANES 2007-2016 (Table 5), we observed associations that exhibited similar patterns as the regression results using the subcohort, with some stronger associations. Table 5 shows the number of study participants with given characteristics.

**Table 4:** Twitter-derived neighborhood characteristics and adult health outcomes in the NHANES 2011-2016 subcohort^a^.

Zip code-level Twitter predictors and tertiles		BMI (kg/m^2^), b (95% CI)^b^	Obesity, prevalence ratio (95% CI)^b^	Hypertension, prevalence ratio (95% CI)^b^	Diabetes, prevalence ratio (95% CI)^b^
**Happy tweets**					
	Third tertile (highest)	–0.85 (–1.48 to –0.21)	0.92 (0.82 to 1.04)	0.94 (0.88 to 1.00)	0.90 (0.76 to 1.05)
	Second tertile	–0.65 (–1.10 to –0.20)	0.95 (0.86 to 1.04)	0.92 (0.86 to 0.98)	1.02 (0.90 to 1.15)
**Physical activity tweets**					
	Third tertile (highest)	–0.57 (–1.27 to 0.12)	0.94 (0.85 to 1.04)	0.90 (0.85 to 0.96)	1.09 (0.87 to 1.37)
	Second tertile	–0.18 (–0.83 to 0.47)	1.00 (0.91 to 1.09)	0.92 (0.87 to 0.98)	1.09 (0.91 to 1.32)
**Fast food tweets**					
	Third tertile (highest)	–0.37 (–0.84 to 0.11)	0.98 (0.90 to 1.07)	0.96 (0.88 to 1.04)	1.00 (0.84 to 1.19)
	Second tertile	–0.47 (–1.04 to 0.10)	0.99 (0.89 to 1.10)	0.95 (0.89 to 1.02)	1.00 (0.83 to 1.21)
**Healthy food tweets**					
	Third tertile (highest)	–1.02 (–1.75 to –0.28)	0.88 (0.79 to 0.98)	0.99 (0.91 to 1.06)	1.00 (0.83 to 1.21)
	Second tertile	–0.73 (–1.39 to –0.07)	0.95 (0.86 to 1.04)	0.94 (0.88 to 1.00)	1.00 (0.85 to 1.16)
NHANES participants - 1^c,d^		15,897	15,897	15,412	15,473
NHANES participants - 2^e^		15,774	15,774	15,291	15,353

^a^NHANES 2011-2016 among adults 20 years and older.

^b^Adjusted regression models were run for each outcome. For dichotomous outcomes such as obesity and diabetes (0=no; 1=yes), Poisson models were utilized. For continuous variables like body mass index, linear regression was used. Models controlled for individual-level demographics including age, gender, race/ethnicity, annual household income, as well as zip code–level characteristics such as population density, percent white, median age, and median household income. Twitter-derived characteristics were categorized into tertiles, with the lowest tertile serving as the reference group. Analyses accounted for survey weights and complex survey design to produce nationally representative estimates.

^c^NHANES: National Health and Nutrition Examination Survey.

^d^Number of NHANES participants included in models with zip code–level happy tweets or physical activity tweets as the predictor variable.

^e^Number of NHANES participants included in models with zip code–level healthy food tweets or fast food tweets as the predictor variable.

**Table 5:** Twitter-derived neighborhood characteristics and adult health outcomes in full cohort^a^.

Zip code–level Twitter predictors and tertiles		BMI (kg/m^2^), b (95% CI)^b^	Obesity, prevalence ratio (95% CI)^b^	Hypertension, prevalence ratio (95% CI)^b^	Diabetes, prevalence ratio (95% CI)^b^
**Happy tweets**					
	Third tertile (highest)	–0.79 (–1.25 to –0.33)	0.90 (0.82 to 0.98)	0.94 (0.89 to 0.99)	0.87 (0.77 to 0.99)
	Second tertile	–0.53 (–0.81 to –0.24)	0.93 (0.88 to 0.99)	0.94 (0.89 to 0.98)	0.99 (0.90 to 1.09)
**Physical activity tweets**					
	Third tertile (highest)	–0.69 (–1.19 to –0.19)	0.89 (0.82 to 0.97)	0.91 (0.87 to 0.96)	1.04 (0.87 to 1.24)
	Second tertile	–0.34 (–0.80 to 0.12)	0.95 (0.89 to 1.02)	0.93 (0.89 to 0.97)	1.03 (0.90 to 1.18)
**Fast food tweets**					
	Third tertile (highest)	–0.19 (–0.60 to 0.22)	1.00 (0.93 to 1.08)	0.95 (0.89 to 1.01)	1.05 (0.91 to 1.23)
	Second tertile	–0.26 (–0.71 to 0.18)	1.01 (0.94 to 1.10)	0.96 (0.91 to 1.02)	1.05 (0.90 to 1.23)
**Healthy food tweets**					
	Third tertile (highest)	–1.02 (–1.54 to –0.51)	0.87 (0.80 to 0.94)	0.96 (0.91 to 1.01)	0.93 (0.80 to 1.09)
	Second tertile	–0.80 (–1.26, –0.33)	0.92 (0.86, 0.98)	0.93 (0.89, 0.97)	0.94 (0.83, 1.07)
NHANES participants^c,d^		27,222	27,222	26,151	26,429
NHANES participants^e^		26,814	26,814	25,752	26,029

^a^Data source for health outcome: NHANES 2007-2016 among adults 20 years and older.

^b^Adjusted regression models were run for each outcome separately. For dichotomous outcomes such as obesity and diabetes (0=no; 1=yes), Poisson models were utilized. For continuous variables like body mass index, linear regression was used. Models controlled for individual-level demographics including age, gender, race/ethnicity, annual household income, as well as zip code level characteristics including population density, percent of White, median age and median household income. Twitter-derived characteristics were categorized into tertiles, with the lowest tertile serving as the referent group. Analyses accounted for survey weights and complex survey design to produce nationally representative estimates.

^c^NHANES: National Health and Nutrition Examination Survey.

^d^Number of NHANES participants included in models with zip code–level happy tweets or physical activity tweets as the predictor variable.

^e^Number of NHANES participants included in models with zip code–level healthy food tweets or fast food tweets as the predictor variable.

## Discussion

This study is one of the first to investigate the relationship between Twitter-derived social neighborhood characteristics and individual cardiometabolic outcomes utilizing a nationally representative population. We found that healthy food was associated with lower mean BMI and lower prevalence of hypertension, and Twitter-derived physical activity was associated with a lower prevalence of hypertension. Associations between happy tweets and obesity and diabetes bordered statistical significance in the subcohort analyses (NHANES 2011-2016) but were statistically significant in the full cohort (NHANES 2007-2016). The associations between Twitter-derived characteristics and obesity were more evident in the full cohort than in the subcohort, possibly due to the larger sample size and higher statistical power.

Twitter-derived happiness was associated with lower mean BMI and lower prevalence of obesity and hypertension, suggesting the protective effect of positive emotion on obesity and hypertension. Results have also shown that neighborhoods with high and medium happiness tertiles have similar prevalence of obesity and hypertension, which indicates that the percent happiness in a neighborhood may not have any additional impact on cardiometabolic prevalence once it reaches a threshold. We included both continuous BMI and binary obesity as outcomes. Our study results suggest that higher neighborhood happiness values shift BMI distributions lower. Individuals living in the third tertile have 0.85 kg/m^2^ lower BMI than those living in the lowest tertile of neighborhood happiness. For obesity, this translates to an 8% lower relative risk. Although obesity is clinically important, the result of BMI provided insights for potential community interventions.

In our study, we focused on neighborhood-level happiness derived from Twitter, which is different from individual-level happiness. However, social networks spread happiness, and an individual’s happiness is correlated to that of their neighbors, friends, and families [41]. The influence of affective state on outcomes via health behaviors could explain the link between happiness and a lower prevalence of health outcomes. Prior studies found negative emotions, including anger, depression, and anxiety, as well as stress, were associated with overeating, sedentary lifestyle, and physical inactivity [42-44]. Negative emotions and chronic stress may induce hemodynamic responses that lead to sustained elevation of blood pressure [45]. Although greater happiness is associated with lower cortisol and reduced plasma fibrinogen stress responses, indicating a lower risk for cardiovascular disease [46].

Associations between Twitter-derived physical activity mentions and lower hypertension suggest social learning of physical activity through Twitter may be effective at promoting the prevention of this condition. Health behaviors, including physical activity, occur in clusters rather than independently [47,48]. Information on physical activity and exercise behaviors may spread over the social network [49], and social network users are more likely to exercise if receiving repetitive messages on physical activity [50]. We also found Twitter-derived healthy food was associated with lower mean BMI and lower prevalence of obesity and hypertension. Social learning of healthy eating behaviors may help in shaping eating behaviors and consequently contribute to a lower prevalence of chronic health outcomes. Our results indicate the potential utility of Twitter as a platform to impact chronic disease prevention via behavioral changes.

Although not statistically significant, we observed associations between Twitter-derived social neighborhood characteristics and outcomes in unexpected directions. More fast food tweets were associated with lower mean BMI and lower prevalence of hypertension. Fast food consumption may be less affected by the local food environment but more affected by individual-level characteristics, including gender, socioeconomic status, and personal preferences [23,51-53]. Some fast-food tweets may come from advertisers rather than individual users. Healthy food tweets are generally sent by individual users, which may partially explain why healthy food tweets are significantly associated with certain community-level health outcomes, while fast food tweets are not. We also found a non-significant association between physical activity tweets and the prevalence of diabetes. We postulate that because diabetes is a complex condition affected by both genetics and environmental factors, the disease is unlikely to change swiftly or reflect the effect of the neighborhood environment.

It is important to note that this study is subject to several limitations. While Twitter does not record user demographics, Twitter users are generally younger [54], and there are more male Twitter users than females [55]. Twitter users are not a representative sample of the general population. Nonetheless, we argue that Twitter data, while imperfect, provides useful information about the social environment that corresponds with differentials in health outcomes [56]. In addition, we only collected geotagged tweets that had the latitude and longitude coordinates, representing a small fraction of all publicly available tweets. Thus, geotagged tweets may not fully capture the social environment for all Twitter users. Moreover, our keyword list approach to classification may not capture all tweets that fall within each topic or misidentify irrelevant tweets. However, we anticipate that misclassified tweets will comprise an insignificantly small portion of all tweets. Misclassification could also occur when assigning the sentiment score to a tweet due to the difficulty in recognizing and differentiating sarcastic expressions and humor. We performed validation for sentiment analysis comparing machine-labeled and manually labeled tweets and observed a high agreement between machine and manually labeled data [20].

Additionally, the study is observational and cross-sectional, which inhibits causal inference. We were unable to establish the temporality between Twitter-derived social neighborhood characteristics and cardiometabolic outcomes. To lessen discordance in the time frame and reduce the potential bias introduced from changing social environments, we implemented separate regression analyses for NHANES data from 2011 to 2016 and from 2007 to 2016. Results generally followed the same pattern for the two time periods, and we observed associations between Twitter-derived characteristics with obesity and hypertension.

We did not account for local resources that might influence cardiometabolic outcomes, for instance, the availability of grocery stores and local sources of healthy foods. However, we controlled for zip code-level characteristics, including percent non-Hispanic white, median age, population density, and median household income in the regression analyses.

Our study has several advantages. We utilize a publicly available big data source, allowing us to create neighborhood characteristics for small areas across the entire contiguous United States. This approach differs significantly from the majority of neighborhood studies that are restricted to local geography, given the time-consuming and expensive nature of gathering neighborhood data. Our study advances the use of social media in health research by constructing social neighborhood characteristics and applied these characteristics at individual-level quantitative analyses. Although researchers have been increasingly aware of the value of using social media data in health research, the majority of existing health studies are content analyses. We are not aware of any studies that used quantitative Twitter characteristics in individual-level outcome research. Additionally, leveraging individual data from NHANES allowed us to incorporate objective health assessments and extensive individual-level demographic information.

Our study investigated the relationships between Twitter-derived social neighborhood features and individual cardiometabolic outcomes in a nationally representative population. Our findings show Twitter as an emerging and cost-effective data source for public health that could be used to understand the potential influence of social context on important chronic health conditions. Researchers and public health practitioners may use Twitter as a public health surveillance tool to identify communities with greater risk of cardiometabolic outcomes. Practitioners could also utilize Twitter as a platform for health education and the social promotion of healthy behaviors aimed at reducing the burden for cardiometabolic outcomes.

## Abbreviations

API: application programming interface
MEC: Mobile Examination Center
NCHS: National Center for Health Statistics
NHANES: National Health and Nutrition Examination Survey
RDC: Restricted Data Center
